# Disease management programs in chronic heart failure: Position statement of the Heart Failure Working Group and the Working Group of the Cardiological Assistance and Care Personnel of the Austrian Society of Cardiology

**DOI:** 10.1007/s00508-017-1265-0

**Published:** 2017-10-27

**Authors:** Deddo Moertl, Johann Altenberger, Norbert Bauer, Robert Berent, Rudolf Berger, Armin Boehmer, Christian Ebner, Margarethe Fritsch, Friedrich Geyrhofer, Martin Huelsmann, Gerhard Poelzl, Thomas Stefenelli

**Affiliations:** 1 Department of Internal Medicine 3, University Hospital St. Poelten, Karl Landsteiner Private University, St. Poelten, Austria; 2 Rehabilitation Center, Lehrkrankenhaus der PMU, Pensionsversicherung Grossgmain, Grossgmain, Austria; 3 Department of Internal Medicine, Hospital Hartberg, Hartberg, Styria Austria; 4 Center for Cardiovascular Rehabilitation, Bad Ischl, Upper Austria Austria; 5 Department for Internal Medicine I, Convent Hospital Barmherzige Brueder, Eisenstadt, Burgenland Austria; 6 Department of Internal Medicine 1, University Clinic Krems, Krems, Lower Austria Austria; 7 Department of Internal Medicine 2, Convent Hospital Elisabethinen, Linz, Upper Austria Austria; 8 Working Group for Preventive Medicine (AVOS), Salzburg, Austria; 9 University Clinic of Internal Medicine II, Medical University Vienna, Vienna, Austria; 10 University Clinic of Internal Medicine III, Medical University Innsbruck, Innsbruck, Tyrol Austria; 11 Department of Internal Medicine 1, Donauspital/SMZ Ost, Vienna, Austria; 12 Heart Failure Working Group, Austrian Society for Cardiology, Vienna, Austria; 13 Working Group of the Cardiological Assistance and Care Personnel, Austrian Society of Cardiology, Vienna, Austria; 14 Institute for Research of Ischaemic Cardiac Diseases and Rhythmology, Karl Landsteiner Society, St. Pölten, Austria

This position statement addresses the most relevant issues pertaining to a comprehensive disease management program (DMP) for patients with chronic heart failure (CHF).

1. Why is a DMP needed?
2. How should a program be organized and how should it function?
3. Which professional groups should be involved?
4. Which patients should be included?
5. What should be the duration of patient care in the program?
6. What are the costs and cost-effectiveness?

## Why is a DMP needed?

Heart failure (HF) represents a major public health problem and, despite optimal medical therapy, morbidity and mortality remain high. The prevalence of CHF is estimated to be 1–2% of the adult population in developed countries, rising to 10% and more in persons 70 years and older [1–9]. The prevalence has continuously increased in the past decades and is expected to rise further over the next decades due to demographic trends [10, 11]. The prognosis of patients with CHF is poor and worse than for most types of cancer [12, 13]. Despite remarkable improvements in medical therapy, prognosis still remains alarmingly poor with a 5-year mortality of 40–50% [12, 13]. Post-hospital discharge mortality in particular has not substantially improved over time [14]. Notably, mortality in CHF patients with preserved ejection fraction (HFpEF) is only slightly lower than that of patients with reduced ejection fraction (HFrEF) [15]. Heart failure is the most common diagnosis at hospital discharge in patients over 65 years [16]. In 2015, of the 26,871 patients discharged from Austrian hospitals with HF documented as the main diagnosis, 17 were 14 years old (0.06%), 204 (0.8%) were 15–44 years old, 2114 (7.9%) were 45–64 years old and 24,536 (91.3%) were >65 years old [17].

The readmission rate after discharge from hospital is substantially high with up to 50% of patients being readmitted within 6 months [18–21]. Also, the risk of death is greatest in the early period after discharge [22]. These findings suggest a role for increased surveillance in the early post-discharge period of greatest vulnerability after HF admission. The treatment of CHF is expensive and industrialized countries spend 2–4% of their annual healthcare budget exclusively on this disease [23]. If these percentages are extrapolated to the Austrian healthcare system, the annual expenditure on CHF could be estimated to be approximately 350 million €. Given that approximately two thirds of HF healthcare expenses are due to in-hospital treatment, repeat hospitalization substantially contributes to the enormous overall economic burden of the disease [24]. Estimates indicate that up to two thirds of HF readmissions are triggered by potentially preventable factors, including suboptimal discharge planning, non-adherence to heart failure medication, inadequate follow-up, insufficient social support, and delays in seeking medical attention [25–27].

Post-discharge disease management programs have been established to prevent readmission, and reduce mortality and healthcare costs. A number of randomized controlled trials of multidisciplinary managed care versus usual care and meta-analyses indicate a reduction of hospitalization and mortality and improvement in cost-effectiveness [28–34]. The vast majority of these trials have concentrated on patients who have had a recent hospital admission for heart failure.

A recent systematic review of 47 trials took into account the heterogeneity in models of care used in different studies: multiprofessional HF clinics, multiprofessional follow-up without HF clinics, telephone contact, primary care follow-up, and enhanced patient self-care [35]. Home visit programs and clinic-based multidisciplinary programs reduced all-cause readmission within 3–6 months by 25% and 30%, respectively. Mortality rates in this period were reduced by 23%, and 44%, respectively. Also in this analysis structured telephone support reduced mortality by 31%.

Based on this evidence, the European Society of Cardiology (ESC) strongly recommends (recommendation class I, level of evidence A) that HF care be provided in a multidisciplinary program [36].

A 3-arm trial in 278 CHF patients conducted in Austria showed that N‑terminal pro‑B-type natriuretic peptide (NT-proBNP) guided, nurse and hospital-led patient management on top of multidisciplinary care is cost-effective and can further reduce all-cause mortality and heart failure hospitalizations [37]. Despite compelling evidence in favor of DMPs, of the various regional DMPs for patients with HF initiated in Austria over the last decades, only a few remain active. Currently, Austria urgently needs but still lacks a nationwide approach to provide structured disease management for CHF patients.

## How should a DMP for heart failure be organized and how should it function?

The ESC guidelines on the management of HF give disease management programs the highest level of recommendation and evidence (I A) and specify characteristics and components of DMPs for HF (Table 1; [1]). More detailed standards for the management of CHF have also been recently published by the ESC Heart Failure Association [38].

**Table 1:** Characteristics and components of a DMP for CHF patients [1]

*Characteristics*
Should employ a multidisciplinary approach (e.g. cardiologists, primary care physicians, nurses, pharmacists)
Should target high-risk symptomatic patients
Should include competent and professionally educated staff
*Components*
Optimized medical and device management
Adequate patient education, with special emphasis on adherence and self-care
Patient involvement in symptom monitoring and flexible diuretic usage
Follow-up after discharge (regular clinic and/or home-based visits; possibly telephone support or remote monitoring)
Increased access to healthcare (through in-person follow-up and by telephone contact; possibly through remote monitoring)
Facilitated access to care during episodes of decompensation
Assessment of (and appropriate intervention in response to) an unexplained increase in weight, nutritional status, functional status, quality of life and laboratory findings
Access to advanced treatment options
Provision of psychosocial support to patients and family and/or caregivers

As shown, a number of randomized controlled trials have successfully tested various types of DMPs in HF patients. A number of randomized controlled trials of multiprofessional, organized or managed care vs. usual care have been carried out [28–32] employing different approaches, such as multiprofessional HF clinics, multiprofessional follow-up without CHF clinics, telephone contact, primary care follow-up, and enhanced patient self-care. A systematic review of 29 such trials found a reduction of mortality by 25%, of CHF hospitalizations by 26% and of all-cause hospitalizations by 19% [39]. Another systematic review and meta-analysis found that case management type interventions led by a HF specialist nurse reduce readmissions and all-cause mortality [40]. A common component of all these interventions was telephone support by a HF specialist nurse. Similarly, home-based nursing care and structured telephone support seem to be the best strategies to prevent readmissions according to another recent review [35]. Comprehensive discharge planning plus post-discharge support for older patients with CHF significantly reduces readmission rates and may improve health outcomes, such as survival and quality of life (QoL) [41].

One of the main tasks of a DMP is to address the complexity of care of heart failure patients. Contributing factors to this include the average age at diagnosis (76 years old), multiple comorbidities of the disease and side effects of drug treatment. Moreover, most patients have numerous medical contacts across all sectors of care, which, if not properly organized, jeopardizes continuity of care and causes suboptimal treatment with adverse outcomes. When hospitalized, patients are admitted to a variety of departments including cardiac care units, intensive care units, emergency rooms, cardiology wards, and internal wards. In outpatient settings, patients are seen by general practitioners (GPs), specialists for internal medicine, cardiologists, or heart failure specialists in hospital-based outpatient units. Due to their comorbidities, patients are also seen by other specialists such as endocrinologists, nephrologists, pulmonologists, and neurologists. Disease management also becomes more complex as new evidence emerges, associated comorbidities increase and the number of treatment recommendations grows.

The goals of a DMP are to provide evidence-based diagnosis and therapy for patients with heart failure and to educate patients and their caregivers. The overall aim is to improve symptoms and QoL while reducing hospitalizations and mortality. Although many DMP have features tailored to local circumstances, essential components of successful DMPs include:

1. multidisciplinary involvement of specialist HF cardiologists and specialist HF nurses,
2. integration of all sectors of care,
3. HF outpatient clinics,
4. adherence to guidelines.

Core aspects of a DMP include the creation of a network between hospital and outpatient structures, the establishment of a seamless system of care in the outpatient setting, the education of patients and improvements in adherence. Optimization of disease-modifying HF therapy is an essential task of a DMP. The need for titration of CHF therapies usually extends beyond the length of the hospital stay; therefore, it is vital to have effective information management between hospital and office-based physicians and nurses on the one side and patients and caregivers on the other.

Agreement on and adherence to common guidelines is crucial for an effective DMP. All patients must be guaranteed evidence-based drug treatment, and for those eligible, standardized access and referral pathways for non-surgical device therapy, such as cardiac resynchronization therapy (CRT), implantable cardioverter defibrillator (ICD), mechanical support (ventricular assist devices), heart transplantation, physical training/rehabilitation and palliative care.

Hospital-based HF outpatient units play a key role in DMPs. Usually directed by a cardiologist specialized in HF in cooperation with a HF nurse, outpatient units serve as a referral center for the entire network. The hospital-based HF cardiologist usually acts as the central contact person for many of the major decisions including those involving HF devices and ventricular assist devices, heart transplantation, management of the most complex cases, and (in cooperation with other players) the establishment of guideline-based management algorithms and standards within the network. The HF outpatient units should provide a supportive milieu for those involved in the care of HF patients and act as a forum for discussions, advice, and training programs especially designed for health care professionals. These outpatient units serve as rapid access points which provide HF expertise to primary and secondary care physicians, other specialist healthcare professionals, and patients [38].

Patient monitoring is a key function of a DMP to support up-titration of HF medication, early recognition of worsening HF, documentation and quality control, and feedback for patient education. Apart from routine clinical follow-up, little evidence exists that favors one specific monitoring method over another. Nevertheless, current trends seem to indicate that the HF management of the future will involve some kind of telemonitoring [33, 42–45]. Remote monitoring of indicators of deterioration via telemonitoring and structured telephone support may help to prevent emergency admissions and to avoid complications. Clear standards are necessary to define which parameters need to be monitored at what time points and by whom. Set guidelines should also determine the appropriate responses to incoming data and decide who should be responsible for monitoring the response effectiveness.

In addition, it is important for a DPM to establish algorithms and standards for troubleshooting. Patients, caregivers and treating physicians must know what to do and who to contact in case of emergency problems or worsening HF symptoms. Furthermore, the DMP should provide patient education, which includes self-empowerment and self-management. Programs teach patients how to control their body weight, measure blood pressure and heart rates, adhere to medication regimes, and recognize symptoms. They also stress the importance of regular physician visits. Self-management programs also teach patients how to interpret obtained variables and react accordingly. Table 2 shows various aspects of patient education according to the ESC guidelines.

**Table 2:** Key topics and self-care skills to include in patient education and the professional behavior to optimize learning and facilitate shared decision making. (Adapted from [36])

Education topic	Patient skills	Professional behavior
Definition, etiology and trajectory of HF (including prognosis).	Understand the cause of HF, symptoms and disease trajectory. Make realistic decisions including decisions about treatment at end-of-life.	Provide oral and written information that takes into account educational grade and health literacy of patients. Recognize HF disease barriers to communication and provide information at regular time intervals. Communicate in a sensitive manner information on prognosis at time of diagnosis, during decision making about treatment options, during changes in the clinical condition and on patient request.
Symptom monitoring and self-care	Monitor and recognize change in signs and symptoms. Know how and when to contact a healthcare professional. In line with professional advice, know when to self-manage diuretic therapy and fluid intake.	Provide individualized information to support self-management such as: in the case of increasing dyspnea or edema or a sudden unexpected weight gain of >2 kg in 3 days, patients may increase the diuretic dose and/or alert the healthcare team. Self-care support aids, such as dosette box when appropriate.
Pharmacological treatment	Understand the indications, dosing and side effects of drugs. Recognize the common side effects and know when to notify a healthcare professional. Recognize the benefits of taking medication as prescribed.	Provide written and oral information on dosing, effects and side effects.
Implanted devices and percutaneous/surgical interventions	Understand the indications and aims of procedures/implanted devices. Recognize the common complications and know when to notify a healthcare professional. Recognize the importance and benefits of procedures/implanted devices.	Provide written and oral information on benefits and side effects. Provide written and oral information on regular control of device functioning, along with documentation of regular check-up.
Immunization	Receive immunization against influenza and pneumococcal disease.	Advise on local guidance and immunization practice.
Diet and alcohol	Avoid excessive fluid intake. Recognize need for altered fluid intake such as: increase intake during periods of high heat and humidity, nausea/vomiting. Fluid restriction of 1.5–2 l/day may be considered in patients with severe HF to relieve symptoms and congestion. Monitor body weight and prevent malnutrition. Eat healthily, avoid excessive salt intake (>6 g/day) and maintain a healthy body weight. Abstain from or avoid excessive alcohol intake, especially for alcohol-induced cardiomyopathy.	Individualize information on fluid intake to take into account body weight and periods of high heat and humidity. Adjust advice during periods of acute decompensation and consider altering these restrictions towards end-of-life. Tailor alcohol advice to etiology of HF, e. g. abstinence in alcoholic cardiomyopathy. Normal alcohol guidelines apply (2 units per day in men or 1 unit per day in women) where 1 unit is 10 ml of pure alcohol (e. g. 1 glass of wine, 0.3l of beer, 1 measure of spirits).
Smoking and recreational substance use	Stop smoking and usage of recreational substances.	Refer for specialist advice for smoking cessation and drug withdrawal and replacement therapy. Consider referral for cognitive behavioral theory and psychological support if patient wishes support to stop smoking.
Exercise	Undertake regular exercise sufficient to provoke mild or moderate breathlessness.	Advice on exercise that recognizes physical and functional limitations, such as frailty, comorbidities. Referral to exercise program when appropriate.
Travel and leisure	Prepare travel and leisure activities according to physical capacity. Monitor and adapt fluid intake according to humidity (flights and humid climate). Be aware of adverse reactions to sun exposure with certain medication (such as amiodarone). Consider effect of high altitude on oxygenation. Take medicine in cabin luggage in the plane, carry list of treatments and the dosage with the generic name.	Refer to local country specific driving regulations regarding ICD. Provide advice regarding flight security devices in presence of ICD.
Sleep and breathing	Recognize sleeping problems and HF sleep-related issues and how to optimize sleep.	Provide advice such as timing of diuretics, environment for sleep, device support. In presence of sleep-disordered breathing provide advice on weight reduction/control.
Sexual activity	Be reassured about engaging in sex, provided sexual activity does not provoke undue symptoms. Recognize problems with sexual activity, their relationship with HF and applied treatment and treatment of erectile dysfunction.	Provide advice on eliminating factors predisposing to erectile dysfunction and available pharmacological treatment of erectile dysfunction. Refer to specialist for sexual counselling when necessary.
Psychosocial aspects	Understand that depressive symptoms and cognitive dysfunction occur more frequently in people with HF, and that they may affect adherence. Recognize psychological problems which may occur in the course of disease, in relation to changed lifestyle, pharmacotherapy, implanted devices and other procedures (including mechanical support and heart transplantation).	Regularly communicate information on disease, treatment options and self-management. Involve family and caregivers in HF management and self-care. Refer to specialist for psychological support when necessary.

*HF* heart failure, *ICD* implantable cardioverter defibrillator

### Implementation

Successful implementation of a DMP requires:

1. integration into existing structures of the healthcare system,
2. adherence to a clear and transparent implementation process,
3. planning that prevents work overload for certain groups of healthcare professionals,
4. regular training programs for personnel,
5. a high rate of acceptance from healthcare professionals, cost bearers, patients and caregivers,
6. cost-effectiveness.

### Quality control

Evaluation of a DMP is essential. Quality control is based on a defined set of data, which must be obtained to audit quality of care, to assess the implementation of changes in a program and to allow trends to be evaluated. These data sets could form a database for entry and verification of data and may even evolve into an electronic patient record [34, 38]. Such variables comprise organizational aspects of the DMP, such as competencies and staff training. They also include monitoring readmission rates, death rates, device implantation rates, referral times, and patient-based aspects. Patient-based aspects can include achievement of target doses of medication, attainment of patient goals, improvement of QoL, and promotion of self-care. Tools are currently available to assess symptoms, QoL [46, 47] and HF patient self-care abilities [48]. Since depression is a common comorbidity in CHF which increases patient mortality and limits individual success rates within a DMP [49], all DMPs should consider including assessments of depression as well.

## Which professional groups should be involved?

A multidisciplinary approach is essential for a DMP.

Key players are:

- cardiologists with special interest and expertise in HF in a tertiary or secondary care center,
- cardiologists, specialists for internal medicine or general practitioners with special interest in HF in primary care,
- HF specialist nurses.

These key individuals must have access to specialists in various fields of medicine, such as nephrologists, diabetologists and neurologists. The inclusion of allied health care professionals, such as pharmacists, physiotherapists, psychologists, and social workers can also be beneficial.

The ESC Heart Failure Association (HFA) recommends that 25% of the cardiologists in tertiary care centers have a HF remit. The target goal should be 1 HF specialist per 100,000 inhabitants [38]. Inclusion of primary care physicians in multidisciplinary teams is paramount given that a substantial proportion of patients with HF have only limited access to a cardiologist or a specialist for internal medicine with HF expertise. This is particularly true in rural areas. Regular training by expert cardiologists in HF best practice methods is essential for involved physicians.

The value of graduate nurses specialized in CHF for reducing hospitalizations due to decompensated CHF has been confirmed in numerous studies [28–30, 32, 50]. The ESC HFA has set a target of one specialized CHF nurse per 100,000 inhabitants [38]. Depending on the structure of the DMP, the potential functions of such nurses could include: conducting home visits, maintaining telephone contacts, facilitating telemonitoring or a combination of these. The main focus should be on patient education and counselling, monitoring of therapy optimization, and recognition of impending deterioration of clinical status in patients. The HF nursing services could function as a key link between secondary and primary care [38]. In practical terms, a home care nurse as part of a DMP needs to be specially trained in CHF care and decision making. Training needs to be structured and should be accredited by qualified authorities. Until such educational programs can be implemented, transitional arrangements may be necessary.

The CHF nurse training programs should include:

- In-depth training in HF, it’s causes, natural history, prevention, diagnostics, evidence-based treatments for individual patients according to guidelines including pharmacological and non-pharmacological therapy, devices and surgery with a special emphasis on drug titration.
- Competency training in performance of clinical assessments and evaluation of symptoms and effects of treatment.
- Competency training in assessment of educational and psychosocial needs and providing patient education.

Inpatient or outpatient cardiac rehabilitation centers should be part of DMPs. Initiation of physical training, structured patient education and optimization of medical therapy are cornerstones of the rehabilitation process [1, 51]. Rehabilitation centers could also play a role in recruiting DMP patients. Rehabilitation programs for HF have been shown to improve QoL and may even reduce patient prehospitalization and mortality [52, 53]. Finally, a coordinator is helpful to manage the activities of key players and ensure the efficient cooperation of all involved partners. In nurse-led DMPs, the HF nurse also typically acts as the coordinator.

## Which patients should be included in a DMP?

According to the ESC guidelines, DMPs for CHF patients should target high-risk patients [1]. The high-risk patient population basically comprises the following patient groups:

1. Hospitalized patients with CHF due to a high risk of readmission after discharge. Inclusion of these patients must occur as part of a discharge plan or immediately following discharge into a DMP in order to provide early follow-up visits.
2. Ambulatory patients at high risk for HF events, especially hospitalization and death. This group includes patients who can be stabilized and gain a marked improvement in prognosis through intensified care before hospitalization becomes inevitable.
3. Patients who are reaching the end of life. At this stage much of the suffering (and costs) could be avoided by seamless transmission of health information and a clear management strategy within a network. Investigating this approach in a trial is challenging due to the difficulty to prospectively define an end-of-life HF population; therefore, data supporting such an approach are still lacking.

Typical HF patients undergo a patient journey pattern seen in a study of more than 8000 Canadian CHF patients [20, 54]. According to this analysis there are two peaks of hospitalization frequency: one early after discharge, which accounts for about 30% of all readmissions and corresponds to patient group 1, the other one late in life with a high risk of dying within 2–3 months, which accounts for about 50% of all readmissions and corresponds to group 3.

While group 1 is clearly defined (recent heart failure hospitalization), strict criteria to select patients from groups 2 and 3 are more difficult to determine. Moreover, all these patient groups are very heterogeneous and further risk stratification might optimize selection of candidates with most potential benefits from a DMP. Various methods, such as risk scores can help for risk stratification. Especially natriuretic peptide levels have been proven as powerful single prognostic markers in heart failure. For example, a high NT-proBNP at discharge is highly predictive of death or readmission and there is also a significantly better hospital-free survival in patients who experience a decrease in NT-proBNP compared to those with an increase [55]. Based on clinical experience and available data for inclusion in a DMP [37] we propose a cut-off of NT-proBNP 1500–2000 pg/ml at discharge after HF hospitalization to define a population at high-risk for readmission. Furthermore, NT-proBNP as a criterion for inclusion and guidance of care in a DMP has already been proven to be clinically beneficial and cost-effective in the Austrian healthcare system [37, 56].

## What should be the duration of patient care in the program?

For patients eligible for a DMP, structured care should commence as soon as possible. This means that for patients hospitalized for HF, structured care should be initiated during hospitalization, include the formulation of a treatment plan, and be continued after discharge as recommended by guidelines [36]. Accordingly, ambulatory patients at high risk for hospitalization and/or death should be included in the DMP without delay.

There are no clear recommendations on the duration of patient participation in a HF DMP. Influential factors are patient characteristics, design of and tools used in the DMP, and availability of resources. For example, a DMP providing highly intensified care with a high amount of utilization of healthcare professionals will, for economic reasons, cover only a certain period of time when the patient has such a particular need. On the other hand, if implantable monitoring devices are utilized, patient monitoring for the duration of device usage might be desirable depending on the available manpower to analyze and react to the transmitted data.

In the scientific literature, the length of time a patient received DMP care varies between 3 and 58 months [45, 57]. The period after HF events, such as hospitalization represents a vulnerable phase in the course of treatment and requires a rapid and more intense follow-up to prevent further events. After the patient has been stabilized and medication optimized, less frequent patient visits are required. Structured programs usually have fixed time periods of intensified care for all participants; however, older patients with more comorbidities, and with more severe HF might require longer periods of intensified care. Therefore, disease management should consider not only entry criteria, but also exit criteria, to determine the moment for de-escalation of patient care and re-entry criteria for taking up more intensive care again. An example of such exit and re-entry criteria could be a certain degree of changes in NT-proBNP values or absolute threshold or a combination of both [37]. Such an approach has been shown to be safe and cost-effective [56, 58]. Another method of individualization of the length of intensified care would be re-evaluation of the patient situation every 3 months. For follow-up of a stable HF patient with optimized medication, visits every 6 months to check medication, symptoms and blood laboratory parameters are recommended. Visits to a HF specialist at least every 12–18 months can guarantee that new advances in medicine are offered to patients in a timely manner [38].

## Costs and cost-effectiveness

An essential component of any successful DMP for CHF patients is its public and sustained funding. Of the CHF costs 60% are attributable to hospitalizations, 13% to nursing homes, 9% to home health care, 9% to medication and 7% to physicians [24]; therefore, any intervention capable of reducing hospitalizations in CHF is very likely to be cost-effective.

An Austrian hospital-based DMP project conducted in Krems, Lower Austria, focusing on up-titration of HF medication in HFrEF patients [59] achieved major cost savings (−1382 € savings per patient over 6 months) through prevention of hospitalization (0.95 hospitalizations less per patient in 6 months), and after an unavoidable admission to hospital, reduced hospital stays by 2 days. In addition, the necessity of implanting devices such as ICDs and CRT has been reduced by about two thirds as a result of the DMP.

The aforementioned Vienna study [37] was also analyzed for cost-effectiveness. The approach using NT-proBNP levels to guide therapy was cost saving compared to usual care, whereas home-based nursing proved to be cost neutral [58]. Furthermore, there was also a cost-utility analysis (CUA) performed based on Austrian as well as on Canadian costs [56]. The NT-proBNP-based approach was not only the most cost-effective approach but also dominant compared to the multidisciplinary home-based nursing care and usual care, thereby not only gaining quality-adjusted life years but also saving money. The probabilities for the NT-proBNP-based approach being the most cost-effective strategy were 92% at a threshold value of 40,000 € for Austria and 93% at a threshold value of Canadian $ 50,000. Contradictory results exist for telemonitoring approaches, although some analyses indicated cost-effectiveness [60, 61].

The most cost-effective therapies, which also have a significant impact on mortality and/or the rate of hospitalization, are disease-modifying drugs, including beta-blockers, angiotensin-converting enzyme (ACE) inhibitors, angiotensin II receptor blockers (ARBs), mineralocorticoid receptor antagonists, ivabradine and sacubitril/valsartan [62, 63]. Implantable devices, such as ICDs and CRT, have also shown advantages despite high initial costs [64]. Therapies such as structured training programs and DMPs that reduce the need for hospitalization and/or improve quality of life, provide at least borderline cost-effectiveness ratios [65]. On the other hand, much more expensive therapies such as mechanical circulatory support with a less clear benefit on survival have very high incremental cost-effectiveness ratios. These factors should be taken into account when a new concept of a DMP is created and initiated. In addition, local availability of resources and community preferences should also be considered. In any case, the most cost-effective strategies, such as optimal drug therapy should be employed first and followed by guideline-recommended device therapy. Reducing HF hospitalizations will accomplish more than saving costs. Given the projected rise in HF hospitalizations in the coming decades, reducing HF hospitalizations will shorten waiting lists for other procedures, limit the number of complex HF cases in hospitals, and alleviate the need for additional hospital beds. Furthermore, a reduction in hospitalizations will also reduce costs in ambulatory post-discharge care. For example, a study in the UK reported that a reduction of hospitalizations by only 10% in a case load of 120,000 patients amounted to savings of £18 million per year [66]. The researchers further estimated that a 40% reduction in hospitalizations by means of a DMP would save the equivalent of annual running costs of the DMP, and if costs of medication (which the patient should be prescribed anyway) were deducted, a 30% reduction in hospitalization would already cover the costs of a DMP. Most readmissions occur within the first 3 months after HF hospitalization; therefore, even if the effect sizes in time to first event analyses of longer studies are lower [67], a 40–50% reduction of such events seems easily achievable with an effective turn-over of patients from an intensified care to a less intensive phase in the DMP.

## Conclusion

A HF disease management program will target primarily patients at high risk. For patients included, a DMP can provide a seamless system of care across all sectors from primary to secondary/tertiary care in a multidisciplinary fashion. The net result of such a DMP will be improved clinical outcome and cost-effectiveness.

This position paper strives to define key elements of a DMP to galvanize stakeholders to implement a nationwide structured HF service available to every HF patient at risk in Austria.
